# A study on the enzymatic properties and reuse of cellulase immobilized with carbon nanotubes and sodium alginate

**DOI:** 10.1186/s13568-019-0835-0

**Published:** 2019-07-22

**Authors:** Li-Juan Li, Wen-Jing Xia, Gui-Ping Ma, Yue-Lin Chen, Yue-Yu Ma

**Affiliations:** 1 Department of Medical Examination, Wulanchabu Medical College, Wulanchabu, 012000 Inner Mongolia China; 2 Department of Biological Engineering, Nanjing Normal University Taizhou College, Taizhou, 225300 JiangSu China

## Abstract

Cellulase has many potential applications in ethanol production, extraction of medicinal ingredients, food, brewing, oil exploration, environmental protection. However, the widespread use of cellulase is limited by its relatively high production costs and low biological activity. Therefore, we studied the enzymatic properties and reusability of cellulase immobilized on multiwalled carbon nanotubes and sodium alginate for the first time. The results showed that the optimum temperature and pH of immobilized cellulase was 40 °C and 3.0, respectively. After 1 month of storage at 4 °C, the enzyme activity of immobilized cellulase dropped to 71.2% of the baseline. Immobilized cellulase was proved to be reusable and maintained ~ 70% of its activity after 7 cycles of repeated use. Versus free cellulase, the immobilized cellulase showed good thermal stability, pH resistance, storage stability and reusability, which could be beneficial in large-scale industrial manufacturing processes.

## Introduction

Agricultural countries, such as China, have many straw crops, most of which are accumulated or incinerated in the soil. These crop wastes are leading to pollution and a waste of resources. One alternative solution is converting the straw into gas or liquid fuel to produce energy and minimize pollution concurrently (Li et al. 2009; Duarte et al. 2013). However, it is currently limited by high production costs, low enzymatic activity and high consumption of cellulase. Immobilized enzymes were allowed the enzyme to be reused in multiple cycles to lower the production costs and overcome such technical bottlenecks (Zhang et al. 2005; Wu and Ma 2008; Tao et al. 2006). Researchers typically immobilize cellulase via covalent crosslinking and physical adsorption (Karimi et al. 2014; Matsuura et al. 2006). Carrier materials chosen to immobilize cellulase were nanoscale materials (Bohara et al. 2016), natural polymers (Andriani et al. 2015), mesoporous materials (Zhang et al. 2016a, b) and magnetic materials (Han et al. 2018); however, high leakage rates were discovered in all these single carrier immobilized enzymes. The leakage rates can be reduced via the introduction of functional groups, but the process is complex and the costs of biocatalysis can also be increased (Pang et al. 2015). Two interesting carrier groups that will be highlighted in this work are carbon nanotubes and sodium alginate.

Since their discovery in 1991, carbon nanotubes have attracted much attention due to their unique structure (Huang and Tsai 2009). They are composed of graphite flakes in cylindrical structure. Single-walled carbon nanotubes (SWCNTs) consist of only one sheet of graphite flake curling around a central axis, while multi-walled carbon nanotubes (MWCNTs) are formed by several graphite sheets curling around a central axis (Zhang et al. 2013; Feng and Ji 2011). MWCNTs have better physical and chemical stability, lower price, easier preparation and lower toxicity than SWCNTs (Rastian et al. 2014; Lee et al. 2006). Carbon nanotube immobilized enzymes have a large surface area, high enzyme content, and superior dispersibility (Apetrei and Apetrei 2015; Pang et al. 2015). In addition, the dimensions of the carbon nanotubes are similar to those of the enzyme, which facilitates an efficient reaction (Janegitz et al. 2011). Finally, they are easy to be separated from the reaction system, along with some other features as reusable, feasible in transportation and storage, stable and economical. These characteristics can potentially increase their commercialization (Mohamad et al. 2015; Ansari and Husain 2012; Wan et al. 2015).

Besides its non-toxicity and low price, Sodium alginate has many practical applications (Guzik et al. 2014). It is a natural polysaccharide formed by the glycosidic bond between β-d-mannuronic acid and α-l-guluronic acid, and it can be extracted from marine plants such as sea vegetables and giant kelp (Abd EI-Ghaffar and Hashem 2013). Ca^2+^ can interact with carboxyl groups to form microspheres with the existence of guluronic acid alginate, demonstrating excellent mechanical strength and flexibility (Ozyilmaz and Gezer 2010; Cheirsilp et al. 2009). That is one of the reasons why it is commonly used as a carrier material for immobilized enzymes.

In this study, cellulase has been immobilized with carriers MWCNTs and sodium alginate. This process can minimize the side effects caused by high leakage rates of single carrier immobilized enzymes. These two carriers are easy to obtain and economical, with an extra benefit of lower costs in biocatalysis. In addition, temperature, pH, stability, reusability and other enzymatic properties of the system were studied to provide a basis for the potential industrial application of immobilized enzymes.

## Materials and methods

### Materials

Trichoderma cellulase (10 U/mg) (Shanghai Ryon Biological Technology Co., Ltd., Shanghai, China); MWCNTs with an outer diameter of 15–30 nm and a length of 1.5 μm (Shenzhen Nanotech Port Co., Ltd., Shenzhen, China); Corn stalks (from Chahar Right Banner Wulanchabu, Inner Mongolia).

### Immobilization of cellulase by MWCNTs and sodium alginate

Cellulase (75 mg) was added to 25 mL of 50 mmol/L pH 5.0 citrate-Na_2_HPO_4_ buffer, followed by 20 mg of MWCNTs. The solution was shaken in a 40 °C water bath at 200 rpm for 3 h (Zhou et al. 2014). Next, 100 mL of 3.5% sodium alginate was added, respectively with continuous mixing. The mixture was drawn into a 5 mL syringe to a height of 20 cm, and 2% CaCl_2_ solution was injected in immediately to form smooth globules. Subsequently, the globules were filtered and the CaCl_2_ solution was replaced to static hardening in a 4 °C refrigerator for 2 h. The globules were filtered out again and the water surface was blotted after another wash step. Finally, the immobilized cellulase was obtained and stored at 4 °C (Zhao et al. 2007).

### Determination of cellulase activity

Carboxymethyl cellulase (CMC) was used as a substrate and was determined by the 3,5-dinitrosalicylic acid (DNS) method (Zhou 2008). First, 1.0 mL of 50 mmol/L citrate-Na_2_HPO_4_ buffer (pH 4.8) and 0.5 mL of 1% CMC were added into a 25 mL graduated test tube. Then, 0.5 mL of enzyme solution was added and the tube was put in a 50 °C water bath for 30 min. 3 mL DNS was added afterward and put in boiling water bath for 5 min. Subsequent to cooling process, distilled water was added to maintain a constant volume of 25 mL. The absorbance of the 3-amino-5-nitrosalicylic acid was detected via colorimetric method at 540 nm (0.5 mL distilled water was used to replace the enzyme solution as blank control). The reducing sugar produced by the hydrolysis of cellulase can reduce DNS to 3-amino-5-nitrosalicylic acid, thus the product is red under alkaline condition and has a maximum absorbance at 540 nm. The optical density is proportional to the reducing sugar content within the linear dynamic range. The reducing sugar content was calculated via a standard glucose curve equation, and the cellulase activity was calculated by the following formula. The immobilized cellulase activity was determined by replacing 0.5 mL of enzyme solution with 0.5 mL distilled water and immobilized enzyme, the other steps remained the same as the determination of free cellulase activity. Three independent experiments have been performed to obtain the data. The cellulase activity was calculated as:

$$\begin{aligned} {\text{Free}}\;{\text{cellulase}}\;{\text{activity}}\;\left( {\text{U/L}} \right) = \frac{{{\text{reducing}}\;{\text{sugar}}\;{\text{content}}\;\left( {\text{mg}} \right) \times {\text{N}} \times 1000}}{{0.18 \times {\text{t}} \times {\text{V}}}} \hfill \\ {\text{Immobilized}}\;{\text{cellulase}}\;{\text{activity}}\;\left( {\text{U/g}} \right) = \frac{{{\text{reducing}}\;{\text{sugar}}\;{\text{content}}\;\left( {\text{mg}} \right) \times 1000}}{{0.18 \times {\text{t}} \times {\text{g}}}} \hfill \\ \end{aligned}$$

Note: t: reaction time of enzyme and substrate, V: volume of added cellulase solution during the determination of enzyme activity, N: dilution ratio, 0.18: 1 μmol glucose is equivalent to 0.18 mg glucose, g: the amount of added immobilized cellulase.

### Determination of enzyme immobilization yield

The total activity of the free cellulase added during the immobilization process, M_0_, and the activity of cellulase in the supernatant after immobilization, M_1_, were detected. Three independent experiments have been performed to obtain the data. The immobilization yield was calculated as:

$$\left( {{\text{M}}_{0} - {\text{M}}_{1} } \right)/{\text{M}}_{0} \times 100\%$$

### Effect of sodium alginate concentration on cellulase immobilization yield

The cellulase immobilization yield were measured by altering different sodium alginate solutions in the concentration of 2%, 2.5%, 3%, 3.5% or 4%.

### Effect of enzyme concentration on immobilized cellulase activity and immobilization yield

The immobilized cellulase activity and immobilization yield were measured by altering enzyme solution with different concentration as 1 mg/mL, 2 mg/mL, 3 mg/mL, 4 mg/mL, 5 mg/mL, 6 mg/mL.

### Effect of temperature on cellulase activity

The cellulase activity on immobilized and free enzymes were measured by altering water bath temperatures as 30 °C, 40 °C, 50 °C, 60 °C and 70 °C, respectively. The graph was drawn with time being the abscissa and relative cellulase activity and immobilized cellulase activity being the ordinate.

### Effect of pH on cellulase activity

The activity of immobilized and free cellulases were measured at 50 mmol/L with citrate-Na_2_HPO_4_ buffer of different pH as 3.0, 4.0, 5.0, 6.0 and 7.0 in a 50 °C water bath. The graph was drawn with pH being the abscissa and relative cellulase activity and immobilized cellulase activity being the ordinate.

### PH stability of cellulase

The immobilized and free cellulases were added to 50 mmol/L citrate-Na_2_HPO_4_ buffer of different pH as 3.0, 4.0, 5.0, 6.0 and 7.0 in a 40 °C water bath for 1 h. The cellulase activity was then measured. The initial cellulase activity was 100%, and the trend of cellulase activity changing over time was showed with pH being the abscissa and relative activity and immobilized cellulase activity being the ordinate.

### Storage stability of cellulase

The immobilized and free cellulases were stored in a 4 °C refrigerator for 1 month. The cellulase activity over time was determined with the initial cellulase activity set as 100%.

### Reusable hydrolysis CMC by immobilized cellulase

Immobilized cellulase (0.2 g) was added to a 25 mL graduated test tube, together with 0.5 mL CMC (1%) and 1.5 mL citrate-NaH_2_PO_4_ buffer (50 mmol/L, pH 3.0). After 30 min reaction in a 40 °C water bath, the reaction liquid was removed to confirm the cellulase activity. The immobilized cellulase was rinsed thrice with 50 mmol/L citrate-NaH_2_PO_4_ buffer (pH 3.0). Next, fresh 1% CMC was added and the activity of the immobilized cellulase was measured again. Finally, the reusability of immobilized cellulase was determined after repeated cycles.

### Straw pretreatment

The straw was cut into 2–3 cm sections and ground into a powder to pass through a 40 mesh screen. 10 g dry straw powder was then placed in a flask along with 200 mL of 1% dilute sulfuric acid and the mixture was left for incubation for ~ 12 h. Then dilute acid hydrolysis was performed at 121 °C for 2 h. The residue was filtered, washed with water until neutral, dried, ground into a 40 mesh powder, cooled to room temperature and stored (Yao 2008).

### Reusable hydrolysis straw by immobilized cellulase

2 g of immobilized **c**ellulase and 1 g of straw powder were added into 50 mL citrate-Na_2_HPO_4_ buffer (50 mmol/L, pH 3.0) and then placed in a 40 °C water bath. After the straw was hydrolyzed for 24 h, the supernatant was removed to measure cellulase activity. The immobilized cellulase was washed thrice with citrate-Na_2_HPO_4_ buffer (50 mmol/L, pH 3.0), and then 1 g fresh straw powder and 50 mL citrate-Na_2_HPO_4_ buffer (50 mmol/L, pH 3.0)was added again for hydrolysis. Next, the reusability of the immobilized cellulase was determined after repeated cycles.

## Results

### Effect of sodium alginate concentration on cellulase immobilization yield

**Fig. 1:**
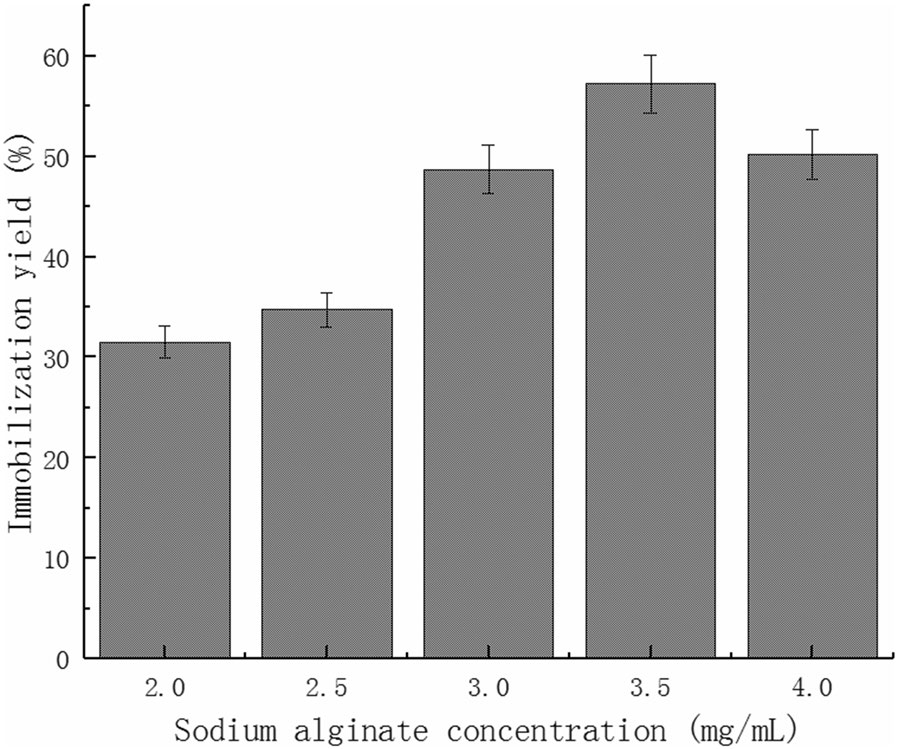
Effects of sodium alginate concentration on cellulase immobilization yield

Figure 1 shows that the immobilization yield of cellulase increases with increasing sodium alginate concentration. The maximum immobilization yield was 57.2% when the concentration of sodium alginate was 3.5%. However, the immobilization yield decreases with further increases of sodium alginate concentration. Thus, the most appropriate concentration of sodium alginate is 3.5%.

### Effect of enzyme concentration on immobilized cellulase activity and immobilization yield

**Fig. 2:**
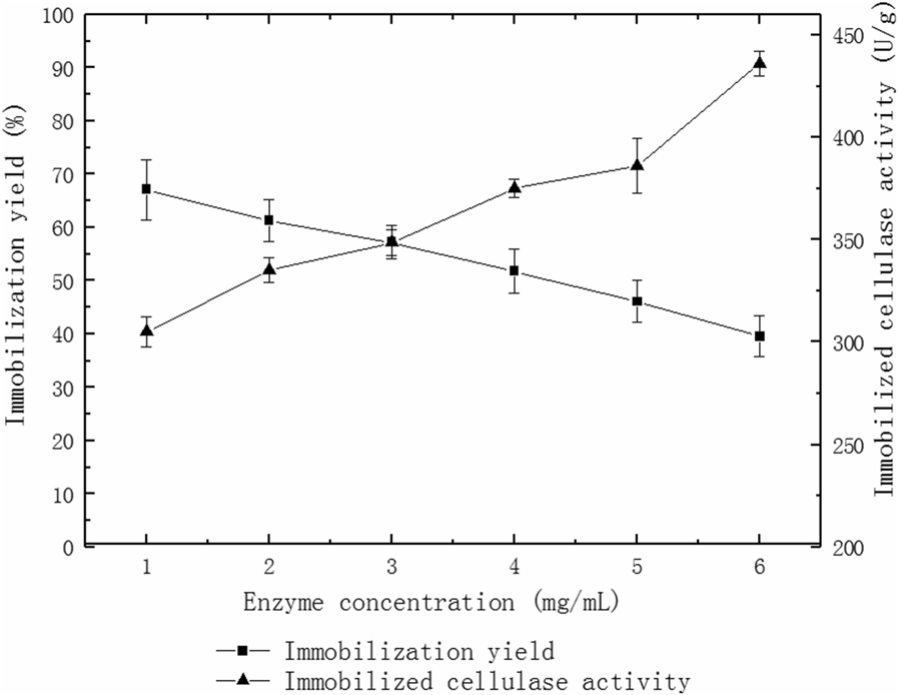
Effects of enzyme concentration on immobilized cellulase activity and immobilization yield

Immobilized cellulase of different concentrations was prepared to study the activity and immobilization yield (40 °C, pH 5.0 citrate-Na_2_HPO_4_ buffer). Figure 2 shows that the activity of immobilized cellulase gradually increases with increasing enzyme concentration while the immobilization yield of cellulase gradually decreases with increasing enzyme concentration. This implies that the enzyme loss rate increases. These observations suggest that an enzyme concentration of 3 mg/mL is the best for further study.

### Effect of temperature on cellulase activity

**Fig. 3:**
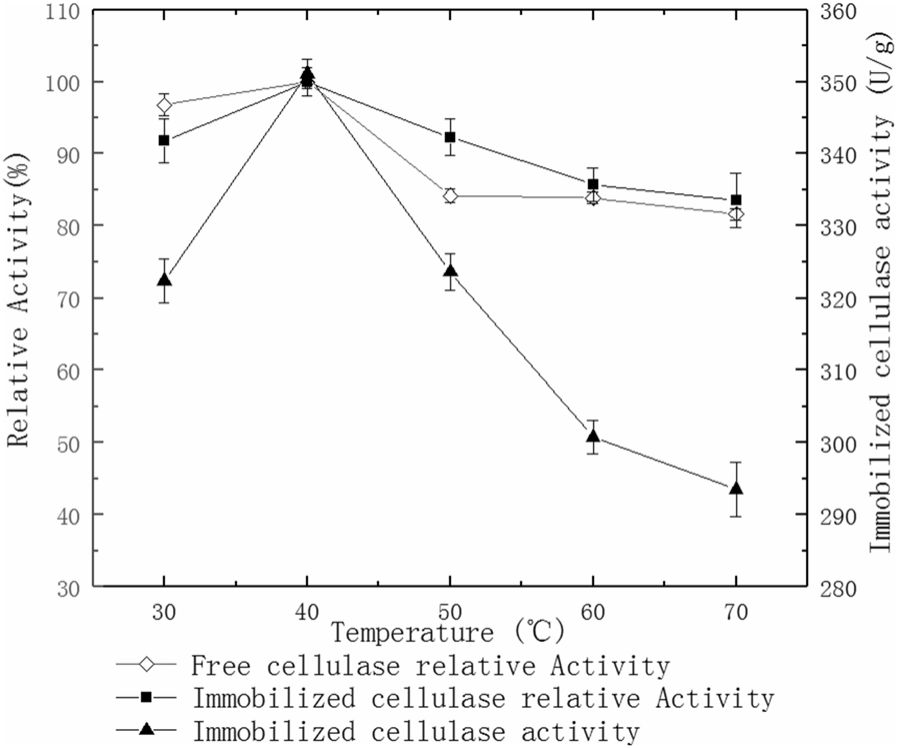
Effect of temperature on cellulase activity

Figure 3 shows that the optimum temperature for both immobilized cellulase and free cellulase is 40 °C. When the temperature is higher, the activity of the immobilized cellulase and free cellulase begin to decrease. The relative activity of the immobilized cellulase is 92.3% at 50 °C. This contrasts with the relative activity of the free cellulase, which is 84.1%. These data confirm that the immobilized cellulase has better thermal tolerance than the free one.

### Effect of pH on cellulase activity

**Fig. 4:**
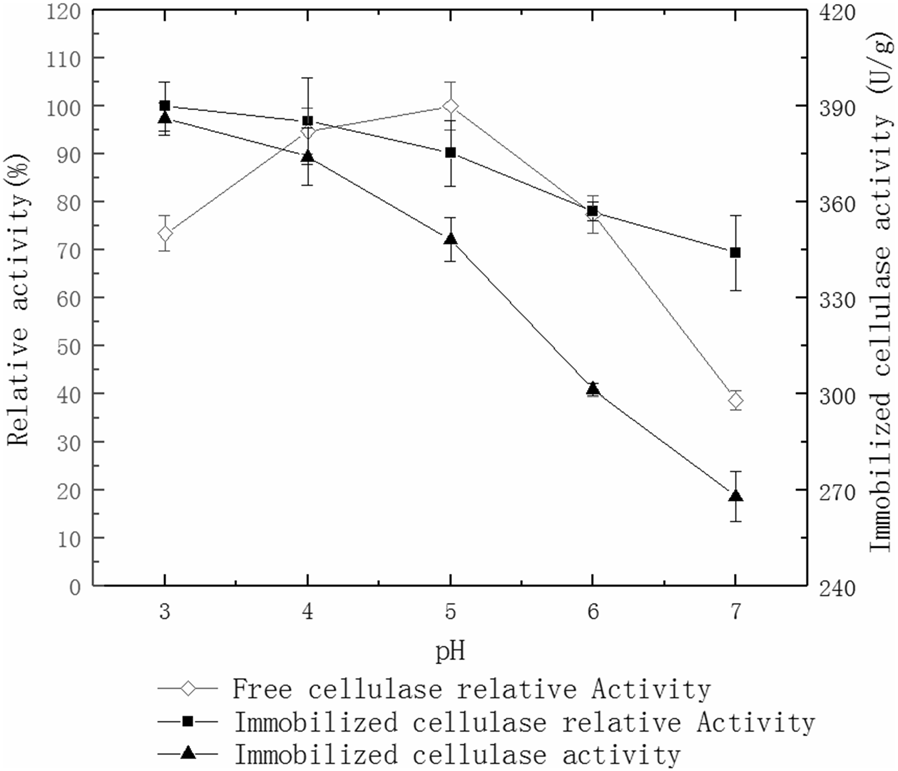
Effect of pH on cellulase activity

The optimal pH of the immobilized cellulase is 3.0 and the free cellulase is 5.0 (Fig. 4). It shows this enzyme works better in acidic conditions.

### Cellulase pH stability

**Fig. 5:**
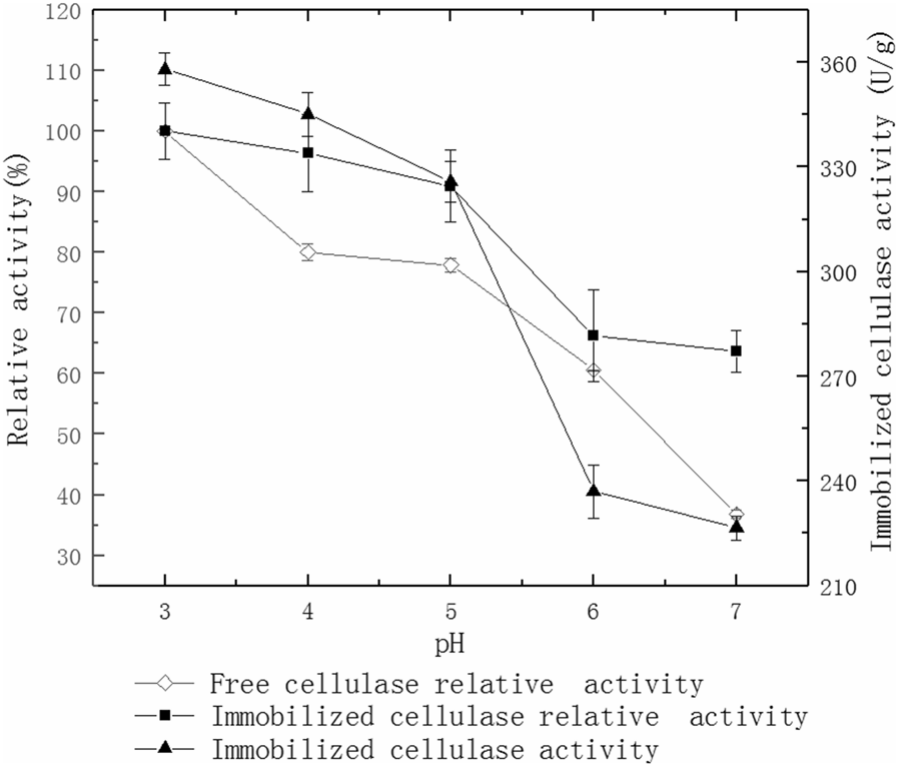
Cellulase pH stability

Figure 5 shows that the best pH for immobilized cellulase and free cellulase to maintain stability is 3.0. Higher pH decreases the stability of cellulase.

### Storage stability of cellulase

**Fig. 6:**
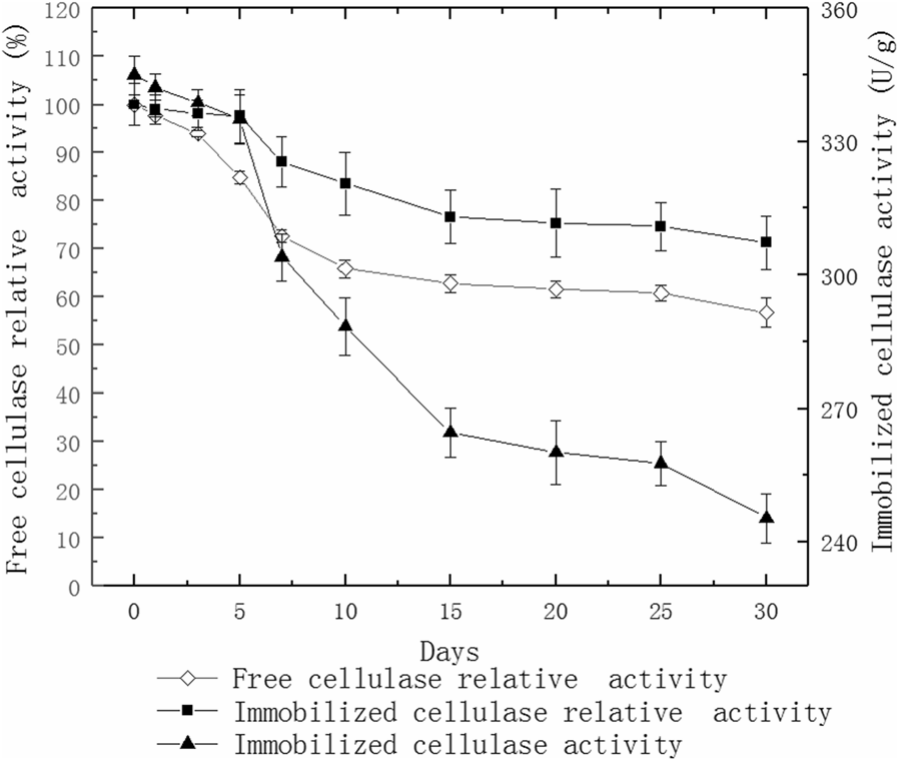
Storage stability of cellulase

Figure 6 shows that the enzyme activity remains at 88.1% of the initial immobilized cellulase activity after it is stored for 7 days; the free cellulase activity decreases to 72.7% of the initial cellulase activity. After storage of the immobilized cellulase for 30 days, the enzyme activity remains at 71.2% of the initial immobilized cellulase activity, but the free cellulase activity decreases to 56.8%.

### Reusable hydrolysis CMC by the immobilized cellulase

**Fig. 7:**
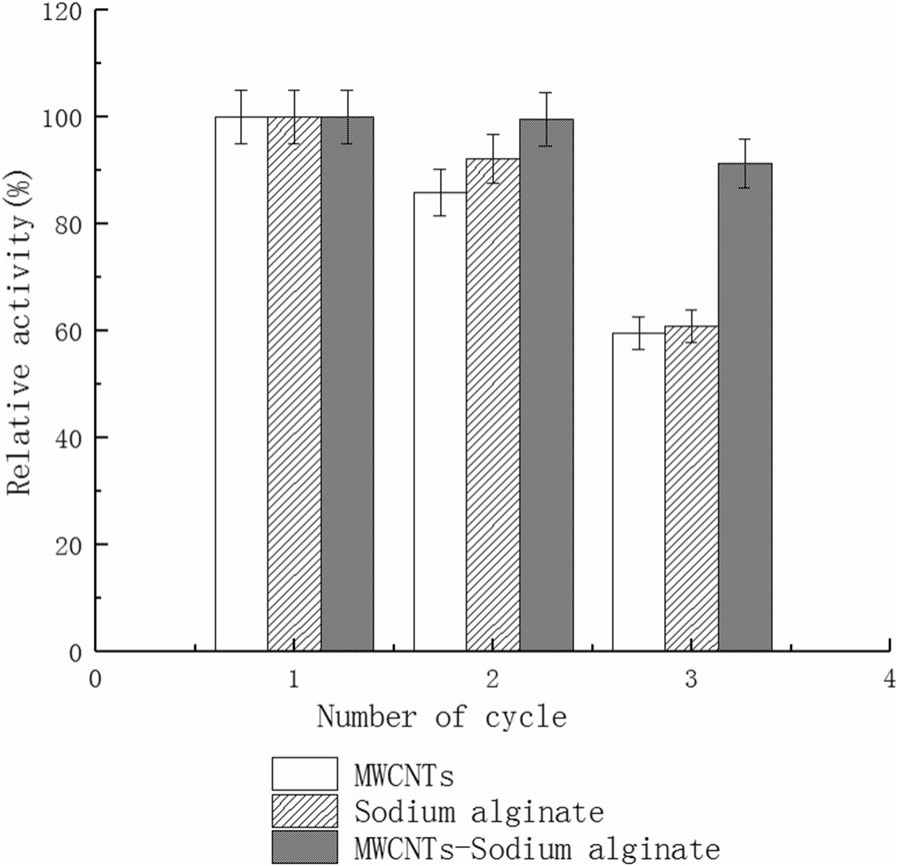
Reusable hydrolysis CMC by the immobilized cellulase of different materials

**Fig. 8:**
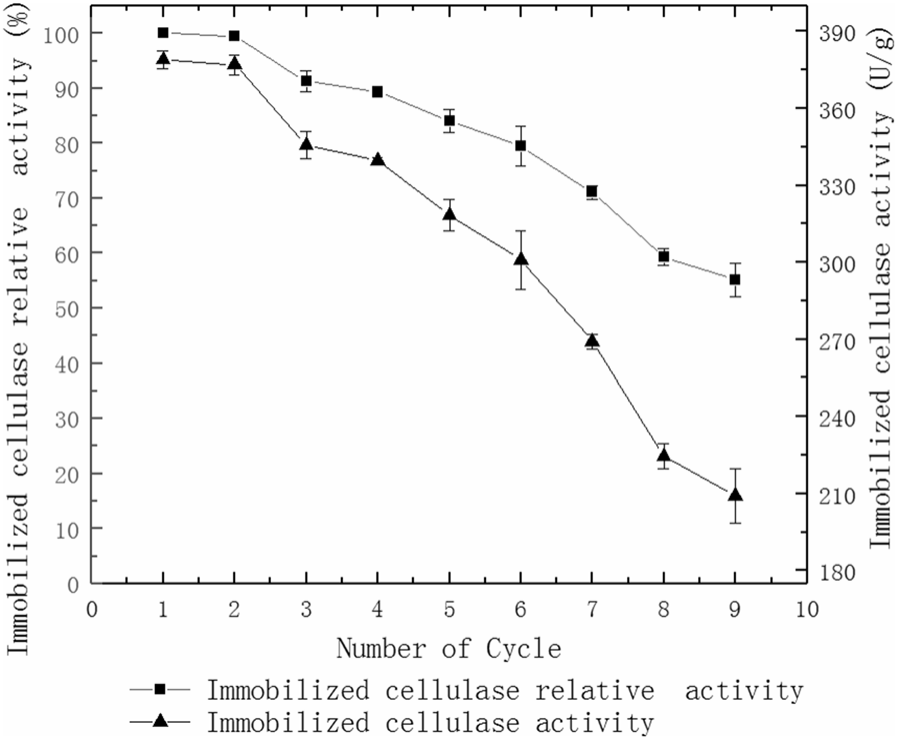
Reusable hydrolysis CMC by the immobilized cellulase

Figure 7 shows that the immobilized cellulase activity is only 59.5% of the initial immobilized cellulase activity when directly immobilized with MWCNTs and after three reused cycles. The immobilized cellulase activity is only 60.9% of the initial immobilized cellulase activity when directly immobilized with sodium alginate and after three reused cycles. The immobilized cellulase activity remains at 91.2% of the initial immobilized cellulase activity when immobilized with MWCNTs and sodium alginate and after three reused cycles. The immobilized cellulase activity remains at 71.5% of baseline after seven reused cycles (Fig. 8) with MWCNTs and sodium alginate.

### Reusable hydrolysis straw by immobilized cellulase

**Fig. 9:**
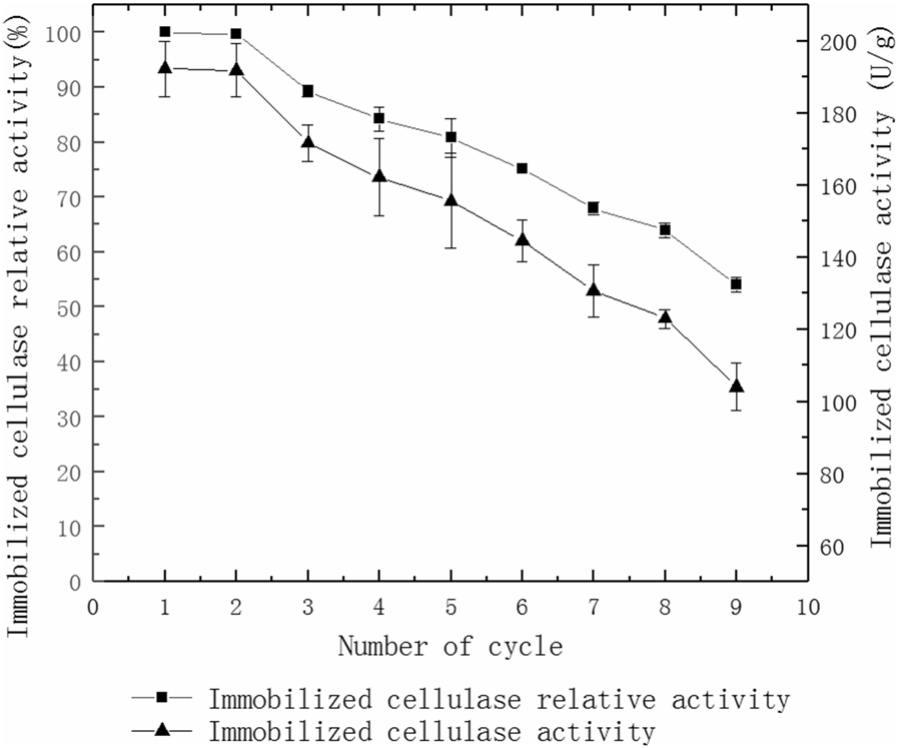
Reusable hydrolysis straw by immobilized cellulase

Figure 9 shows that the immobilized cellulase activity remains at 67.9% of the initial immobilized cellulase activity after seven repeated straw hydrolysis cycles.

## Discussion

Here, we discuss the enzymatic properties of the cellulase immobilized by MWCNTs and sodium alginate. We’ve studied the material’s ability to hydrolyze straw in a reusable way and compared immobilized cellulase with free cellulase.

The concentration of sodium alginate affects the enzyme immobilization yield, the mechanical strength of the beads and the difficulty of pelleting. When the concentration of sodium alginate is 1% and 1.5%, the beads strength is low with an obvious tail phenomenon. When the concentration of sodium alginate is 3.5%, the beads are smooth and round with moderate mechanical strength. Beads are difficult to form at the concentration of 4% because of the high viscosity of the solution, it is difficult to push the syringe.

The activity of immobilized cellulase gradually increases with increasing enzyme concentration (Fig. 2). The increased activity of the immobilized cellulase is due to the large number of pores on the surface of the MWCNTs. These pores could absorb cellulase; the sodium alginate could perform similarly.

The temperature has a dual effect on enzyme activity: The enzyme activity inhibits at low temperature. The enzyme activity denatures at high temperature and their activity decreases. The optimal temperature for immobilized cellulase and free cellulase is of 40 °C. The immobilized cellulase has better thermal tolerance than free cellulase (Fig. 3) because the carbon nanotubes and sodium alginate offer a rigid external backbone for cellulase molecules. Similarly to previous studies, the effect of high temperature on the active center of the enzyme becomes less prominent (Zhang et al. 2016a, b).

In addition, cellulase has a higher activity within pH 3.0 to 5.0. The enzyme activity decreases under alkaline conditions (Fig. 4) because the ionic groups on the surface of cellulase have electrostatic repulsion. This leads to changes in the cellulase activity center and will reduce the overall enzyme activity. However, the activity of immobilized cellulase decreases in a slower speed than that of free cellulase, suggesting a better pH tolerance in enzyme immobilization. These conclusions are similar to previous studies (Liu et al. 2013; Mubarak et al. 2014; Kim et al. 2017). PH of 3.0 offers the best stability for immobilized and free cellulase (Fig. 5). The stability of the cellulase gradually decreases with increasing pH. However, the rate of decrease is lower with immobilized cellulase because cellulase could be packed into the pores of the MWCNTs and embedded in the calcium alginate microspheres. Therefore, the immobilized cellulase has higher pH stability than free cellulase, which is confirmed by other studies (Boncel et al. 2013).

The cellulase activity, respectively remains at 71.2% and 56.8% of the initial cellulase activity after 30-day-storage of the immobilized cellulase and free cellulase (Fig. 6). This indicates that the storage stability of cellulase is significantly improved after immobilization, regarded as a vital prerequisite for industrial applications. Thus, immobilized cellulase can be used for large-scale and industrial processes.

The most obvious advantage of immobilized enzymes over free enzyme is that it can be reused (Cheng 2015). In this study, cellulase is immobilized directly by MWCNTs or sodium alginate, but the leakage rate of the cellulase is high (Fig. 7). The leakage rate of the cellulase is reduced due to the adsorption of MWCNTs and the embedded calcium alginate beads. After 7 cycles of repeated hydrolysis of CMC and the straw, the activity of immobilized cellulase still remains at 71.5% and 67.9%, respectively (Figs. 8 and 9).Good reusability can reduce the amount of free cellulase in industrial production and result in lower production costs. The activity of the immobilized enzyme declines gradually with increasing reuse cycles. This may be because of the relatively weak binding forces via non covalent bonds. Some enzyme molecules cannot be effectively absorbed onto MWCNTs (Hasegawa et al. 2016). The decreased activity may also be due to the hydrophilic characteristics of sodium alginate. The size of the pores gradually becomes larger after repeated use, resulting in increased leakage of the enzyme and reduced activity (Verma et al. 2013).

## Data Availability

All obtained data have been included into the manuscript.

## Abbreviations

CMC: carboxymethyl cellulase
SWCNTs: single-walled carbon nanotubes
MWCNTs: multi-walled carbon nanotubes
DNS: dinitrosalicylic acid

## References

[CR1] Abd EI-Ghaffar MA, Hashem MS (2013). Calcium alginate beads encapsulated PMMA-g-CS nano-particles for- chymotrypsin immobilization. Carbohydr Polym.

[CR2] Andriani D, Sunwoo C, Ryu W, Palmer J, Prasetya B, Park DH (2015). Immobilization of cellulase from newly isolated *strain Bacillus subtilis TD6* using calcium alginate as a support material. Bioprocess Biosyst Eng.

[CR3] Ansari SA, Husain Q (2012). Potential applications of enzymes immobilized on/in nano materials: A review. Biotechnol Adv.

[CR4] Apetrei IM, Apetrei C (2015). The biocomposite screen-printed biosensor based on immobilization of tyrosinase onto the carboxyl functionalised carbon nanotube for assaying tyramine in fish products. J Food Eng.

[CR5] Bohara RA, Thorat ND, Pawar SH (2016). Immobilization of cellulase on functionalized cobalt ferrite nanoparticles. Korean J Chem Eng.

[CR6] Boncel S, Zniszczol A, Szymanska K, Mrowiec-Bialon J, Jarzebski A, Walczak KZ (2013). Alkaline lipase from *Pseudomonas fluorescens* non-covalently immobilised on pristine versus oxidised multi-wall carbon nanotubes as efficient and recyclable catalytic systems in the synthesis of Solketal esters. Enzyme Microb Technol.

[CR7] Cheng L (2015). Horseradish peroxidase immobilized on multi-walled carbon nanotubes/cordierite composite carrier and diesel sewage treatment. J B Univ Chem Technol.

[CR8] Cheirsilp B, Jeamjounkhaw P, Aran H (2009). Optimizing an alginate immobilized lipase for monoacylglycerol production by the glycerolysis reaction. J Mol Catal B Enzym.

[CR9] Duarte JC, Rodrigues JAR, Moran PJS, Valença GP, Nunhez JR (2013). Effect of immobilized cells in calcium alginate beads in alcoholic fermentation. AMB Express.

[CR10] Feng W, Ji PJ (2011). Enzymes immobilized on carbon nanotubes. Biotechnol Adv.

[CR11] Guzik U, Hupert-Kocurek K, Marchlewicz A, Wojciezynska D (2014). Enhancement of biodegradation potential of catechol 1,2-dioxygenase through its immobilization in calcium alginate gel. Electron J Biotechnol.

[CR12] Han J, Rong JH, Wang Y (2018). Immobilization of cellulase on thermo-sensitive magnetic microspheres: improved stability and reproducibility. Bioprocess Biosyst Eng.

[CR13] Hasegawa F, Inoue H, Yano S, Yokoyama S, Imou K (2016). Evaluation of cellulase activity in enzymatic hydrolysis residues for efficient enzyme reuse. J Jpn Inst Energy.

[CR14] Huang JL, Tsai YC (2009). Direct electrochemistry and biosensing of hydrogen peroxide of horseradish peroxidase immobilized at multiwalled carbon nanotube/alumina-coated silica nanocomposite modified glassy carbon electrode. Sensor Actuat B.

[CR15] Janegitz BC, Pauliukaite R, Ghica ME, Brett CAM, Fatibello-Filho O (2011). Direct electron transfer of glucose oxidase at glassy carbon electrode modified with functionalized carbon nanotubes within a dihexadecyl phosphate film. Sensor Actuat B.

[CR16] Karimi M, Chaudhury I, Cheng J, Safari M, Sadeghi R (2014). Immobilization of endo-inulinase on non-porous amino functionalized silica nanoparticles. J Mol Catal B Enzym.

[CR17] Kim YS, Lee CJ, Ma JY (2017). Enhancement of active compound, genipin, from Gardeniae Fructus using immobilized glycosyl hydrolase family 3β-glucosidase from *Lactobacillus antri*. AMB Express.

[CR18] Lee YM, Kwon O, Yoon YJ (2006). Immobilization of horseradish peroxidase on multi-wall carbon nanotubes and its electrochemical properties. Biotechnol Lett.

[CR19] Li HY, Zhang ZQ, Li RH (2009). Study on cellulase enzymatic hydrolysis of microwave-acid pretreated corn stalk. J North A&F Univ.

[CR20] Liu XH, Bu CH, Nan ZH (2013). Enzymes immobilized on amine-terminated ionic liquid-functionalized carbon nanotube for hydrogen peroxide determination. Talanta.

[CR21] Matsuura K, Saito T, Okazaki T, Ohshima S, Yumura M, Iijima S (2006). Selectivity of water soluble proteins in single-walled carbon nanotube dispersions. Chem Phys Lett.

[CR22] Mohamad NR, Buang NA, Mahat NA, Lok YY, Huyop F, Aboul NA, Wahab RA (2015). A facile enzymatic synthesis of geranyl propionate by physically adsorbed *Candida rugosa* lipase onto multi-walled carbon nanotubes. Enzyme Microb Technol.

[CR23] Mubarak NM, Wong JR, Tan KW, Sahu JN, Abdullah EC, Jayakumar NS, Canesan P (2014). Immobilization of cellulase enzyme on functionalized multiwall carbon nanotubes. J Mol Catal B Enzym.

[CR24] Ozyilmaz G, Gezer E (2010). Production of aroma esters by immobilized *Candida rugosa* and porcine pancreatic lipase into calcium alginate gel. J Mol Catal B Enzym.

[CR25] Pang R, Li MZ, Zhang CD (2015). Degradation of phenolic compounds by laccase immobilized on carbon nanomaterials: diffusional limitation investigation. Talanta.

[CR26] Rastian Z, Khodadadi AA, Vahabzadeh F, Bortolini C, Dong MD, Mortazavi Y, Mogharer A, Naseh MV, Guo Z (2014). Facile surface functionalization of multiwalled carbon nanotubes by soft dielectric barrier discharge plasma: Generate compatible interface for lipase immobilization. Biochem Eng J.

[CR27] Tao R, Bai XF, Jiang L (2006). New energy of sustainable development in future: production of alcohol fermented by stover. Liquor Mak.

[CR28] Verma ML, Naebe M, Barrow CJ, Puri M (2013). Enzyme immobilisation on amino-functionalised multi-walled carbon nanotubes: structural and biocatalytic characterisation. Plos ONE.

[CR29] Wan XM, Zhang C, Yu DH (2015). Enzyme immobilized on carbon nanotubes. Prog Chem.

[CR30] Wu XQ, Ma CL (2008). Research on ethanol production through corn stalk fermentation. Mod Agric Sci Technol.

[CR31] Yao QL (2008). Selection of Carbon Sources and Optimization of Conditions for Cellulase Production by *Trichoderma reesei*. J N Univ Fores.

[CR32] Zhang CD, Luo SM, Chen W (2013). Activity of catalase adsorbed to carbon nanotubes: effects of carbon nanotube surface properties. Talanta.

[CR33] Zhang DZ, Hegab HE, Lvov Y, Snow D, Palmer J (2016). Immobilization of cellulase on a silica gel substrate modified using a 3-APTES self-assembled monolayer and James Palmer. Springer Plus.

[CR34] Zhang Q, Lu J, Hou L (2005). Research progress of alcoholic fermentation of corn stover. Feed Ind.

[CR35] Zhang YQ, Wang Z, Tang AX (2016). Preparation of lipase immobilized on functionalized carbon nanotubes and its synthesis of biodiesel. J Renew Energy.

[CR36] Zhao LG, Li LJ, Wang P (2007). Immobilization of β-glucosidase by sodium alginate. Chin J Bioprocess Eng.

[CR37] Zhou H, Qu YY, Kong CL (2014). Catalytic performance and molecular dynamic simulation of immobilized C-Cbond hydrolase based on carbon nanotube matrix. Colloid Surfaces B.

[CR38] Zhou Z (2008). Enhancement in hydrolysis of corncob by cellulase with the addition xylanases. J N Univ Fores.

